# Discovering communities in complex networks by edge label propagation

**DOI:** 10.1038/srep22470

**Published:** 2016-03-01

**Authors:** Wei Liu, Xingpeng Jiang, Matteo Pellegrini, Xiaofan Wang

**Affiliations:** 1 Department of Automation, Shanghai Jiao Tong University, Shanghai 200240, China; 2 School of Computer Science, Central China Normal University, Wuhan, Hubei 430079, China; 3 Department of Molecular, Cell and Developmental Biology, University of California, Los Angeles 90055, CA

## Abstract

The discovery of the community structure of real-world networks is still an open problem. Many methods have been proposed to shed light on this problem, and most of these have focused on discovering node community. However, link community is also a powerful framework for discovering overlapping communities. Here we present a novel edge label propagation algorithm (ELPA), which combines the natural advantage of link communities with the efficiency of the label propagation algorithm (LPA). ELPA can discover both link communities and node communities. We evaluated ELPA on both synthetic and real-world networks, and compared it with five state-of-the-art methods. The results demonstrate that ELPA performs competitively with other algorithms.

Many real-world complex systems can be described using a network, such as social^1^, information^2^, and biological^3^,^4^ networks. One of the primary goals of the study of complex networks is the identification of community structure. Community structure is a critical property of complex networks. Although there is no universal definition of community structure, it is widely accepted that a community in a network should have more internal connections than external ones^5^.

In the last decade, many methods have been proposed to detect the community structure of complex networks. However, most of these approaches focused on the identification of *node community*. Examples of theses approaches include modularity optimization^6^,^7^,^8^,^9^, dynamic label propagation^10^,^11^,^12^,^13^, and information-theoretic methods^14^,^15^,^16^. Among them, the dynamic label propagation algorithm is a widely used *node community* detection method. It updates the label of each node based on the current labels of its neighbors in each time step. Many approaches force one node to only belong to a single community, while in real-world networks, overlapping communities are widespread. Several overlapping community detection algorithms have been developed in recent years^17^,^18^,^19^,^20^,^21^,^22^,^23^,^24^. Among them, *link community* algorithms allow the incorporation of overlap information^16^,^25^,^26^,^27^,^28^,^29^. However, in most cases, the qualities of network partitions of *link community* algorithms are not as optimal as those generated by *node community* algorithms.

In this paper, we propose a novel edge label propagation algorithm (ELPA), which combines the natural advantage of *link community* with the efficiency of the label propagation algorithm (LPA). As a result, ELPA can discover both link communities and node communities. The main idea of ELPA is that densely connected edges should form a consensus *link community* based on their edge labels, and each edge (node) should update corresponding edge labels (node labels) at each time step based on the labels of its neighbors. ELPA includes four stages: (I) initialization, (II) edge label propagation, (III) node label propagation and (IV) bridge identification. The initialization step is used to construct all candidate link communities. Edge label propagation is mainly involved in edge clustering, and node label propagation is mainly involved in node clustering. Next, the bridge identification step marks all bridges. Finally, edges are grouped as link communities, and nodes are grouped into node communities based on their edge labels. Furthermore, edges that cross any two communities are marked as bridges. Thus ELPA is relatively simple, stable and parameter free, and is based only on network topology structure and doesn’t require any *a priori* knowledge. To assess the performance of ELPA, we evaluated it on 24 different types of networks, and compared it with five state-of-the-art community algorithms. The results showed that our approach compares favorably to other methods.

## Results

We tested ELPA on both synthetic and real-world networks. For synthetic networks, we tested the classical benchmark proposed by Girvan and Newman (GN)^30^ and the well-known benchmark with overlapping community structure proposed by Lancichinetti, Fortunato & Radicchi (LFR)^31^. As real-world networks have some different topological properties that distinguish them from synthetic ones, we also tested four kinds of real-world networks: social networks, biological networks, online social networks and collaboration networks.

To assess the performance of ELPA, we compared it with three overlapping community methods (Linkcomm^25^, which is the most widely used *link community* detecting method, COPRA^11^, a representative dynamic label propagation method and NeTA^24^, a simple method based on topological properties) and two disjoint community methods (LPA^10^ is a well-established label propagation method and Infomap^15^, which is an excellent information theory method). These methods have some parameters that need to be set. For Linkcomm, LPA and Infomap, we used their default parameters. For COPRA, we set *v* = 2, *repeat* = 1000, and used the best clusters among them. The codes and parameters setting of LPA and Infomap used here are from the library ‘*igraph*’ of R, and those of Linkcomm and COPRA used here are from the released programs by their respective authors. NeTA is a heuristic method, and is parameter free.

### Synthetic Network

We compared the performance of ELPA with Linkcomm, Infomap, LPA, COPRA and NeTA on disjoint and overlapping benchmark networks respectively. For the disjoint benchmark network, we compared them on one GN benchmark and three disjoint LFR benchmarks. The GN network consists of 128 nodes, arranged in 4 groups of 32 nodes each, and the average degree of the network is 16. The parameter settings for the three disjoint LFR benchmarks are as follows: the network size *n* is set to 256, 512 and 1000 respectively, the maximum degree is set to 50, the average degree is set to 8, the minimum community size is set to 6, 10 and 20 respectively, the maximum community size is set to 50, and the mixing parameter is set to 0.1. For the overlapping benchmarks, we tested nine LFR benchmarks. The network size *n* is set to 1000, the mixing parameter is set to 0.1, 0.2, or 0.3, the number of overlapping vertices is set to 10, 50 or 100, the average degree is set to either 5 or 10, and the number of communities each overlapping vertex belongs to is set to either 2 or 3. The remaining parameters that we keep fixed include the maximum degree, which is set to 50, the minimum community size, which is set to 5, and the maximum community size, which is set to 50.

**Figure 1:**
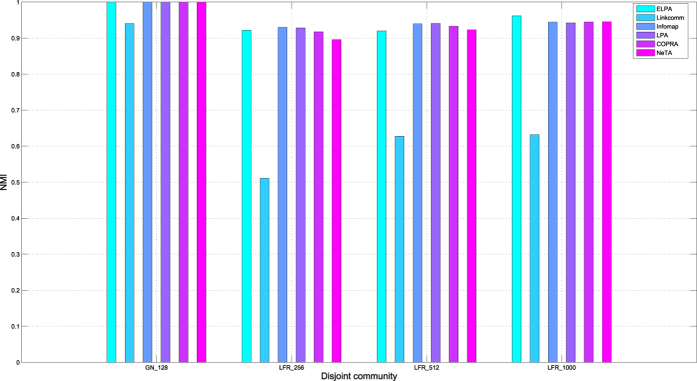
NMI of algorithms on synthetic networks with disjoint communities. Different colors denote different algorithms. The four synthetic networks with disjoint communities are GN, N = 128; LFR, N = 256; LFR, N = 512 and LFR, N = 1000.

Figure 1 shows the results that compare the ELPA method with Linkcomm, Infomap, LPA, COPRA and NeTA on the disjoint benchmarks using NMI as the metric. As we can see, besides Linkcomm, all methods perform with similar accuracy in all four cases. Notice that the Linkcomm method does not perform well here. This is likely because it often finds highly overlapped communities by partitioning links, and fails to detect the communities defined in these benchmarks.

**Figure 2:**
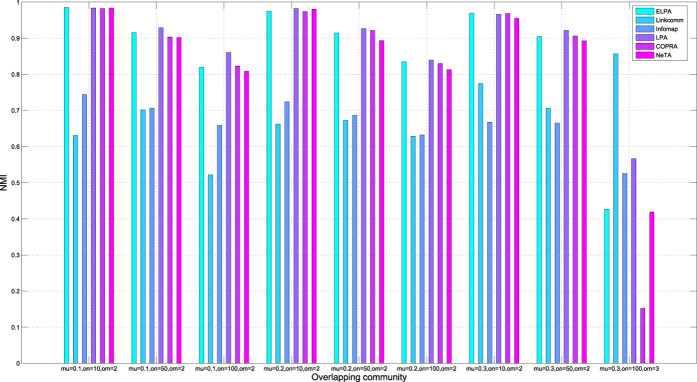
NMI of algorithms on synthetic networks with overlapping communities. Different colors denote different algorithms. The nine synthetic networks with overlapping communities are LFR, mu = 0.1, on = 10, om = 2; mu = 0.1, on = 50, om = 2; mu = 0.1, on = 100, om = 2; mu = 0.2, on = 10, om = 2; mu = 0.2, on = 50, om = 2; mu = 0.2, on = 100, om = 2; mu = 0.3, on = 10, om = 2; mu = 0.3, on = 50, om = 2 and mu = 0.3, on = 100, om = 3 respectively.

Figure 2 shows the results that compare ELPA with five other methods against the overlapping benchmarks using NMI accuracy as the figure of metric. The mixing parameter of these benchmarks is gradually increased from 0.1 to 0.3, and the number of overlapping vertices of these benchmarks is gradually increased from 10 to 100. From the first eight benchmarks we found that the mixing parameter and the number of overlapping vertices have little influence on the accuracy of all methods. ELPA, LPA, COPRA and NeTA perform better than Linkcomm and Infomap, except for the last benchmark. Linkcomm outperforms the other five methods in the last benchmark. Due to the increase in the number of communities each overlapping vertex belongs to, from 2 to 3, the approach performs well on networks with highly overlapped community structure. Based on Figs 1 and 2, we can see that ELPA achieved robust results when applied to both synthetic disjoint benchmark networks and overlapping benchmark networks, compared to other approaches.

### Real-World Networks

The topology structure of real-world networks is more complex than synthetic ones, and it is often hard to know the true structure. Thus, it is still a significant challenge to discover the true network topology. In the following sections we applied ELPA to numerous real-world networks (as shown in Table 1).

#### A Priori network

We report results on four well-known social networks with ground-truth: the Zachary karate club^32^, the American college football^6^, the New Zealand dolphins^33^ and the polbooks^6^. As we have priori knowledge of the disjoint community structures of these networks, we used the NMI measure to evaluate methods. As the same time, we combined NMI with traditional G&N modularity and density measures to further compare the approaches.

**Figure 3:**
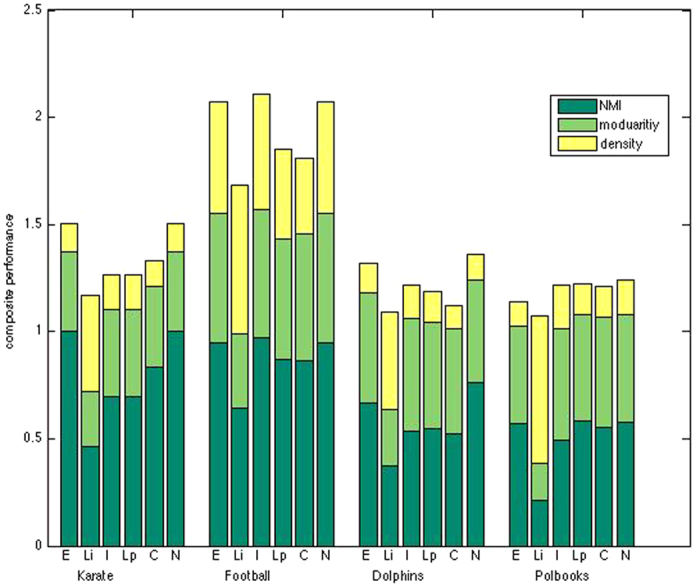
Composite performance of algorithms on four real-world networks with ground-truth. The composite performance including three measurements: NMI, modularity and partition density. The methods are ELPA (E), Lincomm (Li), Infomap (I), LPA (Lp), COPRA (C) and NeTA (N), and the four real-world networks are karate club, football, dolphins and polbooks.

Figure 3 displays the results of the quantitative comparison of methods. We can see that ELPA and NeTA outperform other methods on the karate club, and they all split this club into two disjoint groups, which is consistent with the true internal dissensions of this club^32^. COPRA also found two communities, but slightly different than the true ones. ELPA, Infomap and NeTA outperform other methods on the football network in composite performance, and they correctly identified all the eleven football conferences of Division I-A teams in the fall season of 2000^34^ along with eight independent teams. ELPA and NeTA found eleven communities respectively, while Infomap found 12 communities, and had the highest accuracy based on the NMI measure. The dolphins can be divided into two disjoint groups (one larger and one smaller) based on the long-term observation of researchers^33^. ELPA and NeTA perform better than other methods based on their composite performance. ELPA found four communities. It split the larger one into three small communities, and the remaining community is consistent with the smaller one except for vertex 40. NeTA yielded the best NMI scores, and it found three communities, divided the larger one into two small communities, and the remaining is consistent with the smaller one. The polbooks network is classified into three groups, according to their political preference by Newman^6^. From the composite performance, we can see that Infomap, LPA, COPRA and NeTA perform well, while COPRA found the correct community number, and obtained the best NMI performance. ELPA yielded a better NMI value in all four cases.

From Fig. 3, we can see that ELPA generated high quality results on all four *a priori* networks, whether measured by NMI, modularity, density or the composite performance.

#### A Posteriori Network

We report results on several biological, collaboration and online-social networks that lack a ground-truth, including: Ecoli^35^, netscience^36^, facebook^37^, protein^38^, collaboration^39^, PGP^40^ and twitter^37^. The size of these networks runs from hundreds to tens of thousands. As we don’t *a priori* know the community structures of these networks, overlapping nodes are difficult to evaluate. As a result, we combined overlapping modularity^41^ and partition density^25^ together to improve the reliability of performance measurements.

**Figure 4:**
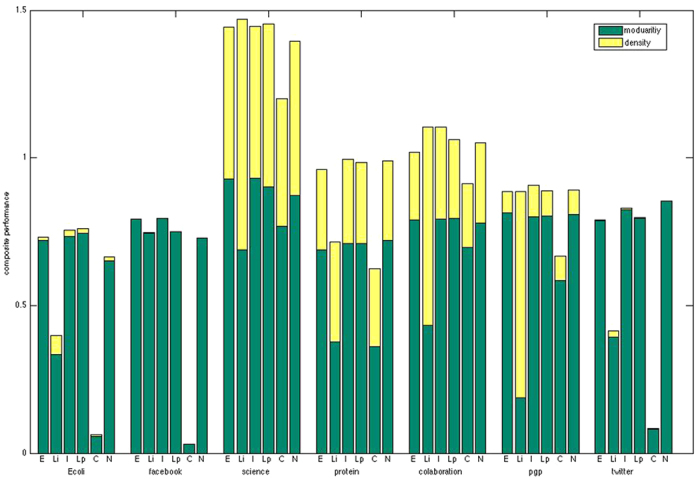
Composite performance of algorithms on seven real-world networks lack of ground-truth. The composite performance including two measurements: overlapping modularity and partition density. The methods are ELPA (E), Lincomm (Li), Infomap (I), LPA (Lp), COPRA (C) and NeTA (N), and the seven real-world networks are Ecoli, net-science, Facebook, protein, collaboration, PGP and twitter respectively.

Figure 4 displays the results of the quantitative comparison of all methods. Infomap and LPA can only detect disjoint communities, while the other four methods can discover overlapping communities. The Linkcomm method outperforms other approaches on all seven networks based on partition density, but it doesn’t obtain higher overlapping modularity on any of these networks. If we only consider the modularity metric, the Infomap method performs best on the Facebook and collaboration networks, the LPA method performs best on the Ecoli and collaboration networks, the NeTA method performs best on the protein and twitter networks, while the ELPA method performs best on the net-science and PGP networks. If evaluated based on the composite performance, the Linkcomm method outperforms other approaches on the net-science network, the Infomap method performs best on Facebook, protein, collaboration and PGP networks, the LPA method performs best on Ecoli network, and the NeTA method performs best on the twitter network. ELPA always performs well on all seven real-world networks based on either overlapping modularity, partition density or composite performance, and its performance compares favorably to the best approah in each case. From this figure, we also note that the COPRA method fails to detect the communities in Ecoli, Facebook and twitter network, and therefore it does not seem suitable for sparse networks when we set *v* = 2.

From Fig. 4 we see that ELPA yields competitive results when run against *a posteriori* real-world networks. In summary, we conclude that ELPA generates stable high quality results for both synthetic and real-world networks, and is competitive with other state-of-the-art algorithms.

## Discussion

Network methods have attracted extensive investigation in recent years. The application of functional module detection methods has been important in many disciplines. Effective community detection algorithms, including *node community* (node partition) methods and *link community* (edge partition) methods, have been proposed during the last decade. *Node community* methods often have higher partition quality than *link community* methods, while link community methods naturally incorporate overlapping communities.

In this paper, we propose a novel edge label propagation algorithm (ELPA), which combines the advantage of link communities and the efficiency of label propagation algorithms (LPA). The advantage of ELPA is its ability to discover both link communities and node communities by using network topology without a priori knowledge. There are two kinds of label propagations in ELPA: one is for edge labels, and the other is for node labels. Edge clustering is processed with unique labeled edges, and link communities are condensed by edge label propagation. We execute node clustering with multiple labeled nodes and node communities are condensed by node label propagation. Most dynamic label propagation algorithms for identifying network community don’t generate stable results. Furthermore, most link community algorithms don’t produce high quality results compared to node community algorithms. By contrast, ELPA generates both stable and high quality results that are competitive with other community algorithms.

ELPA and Linkcomm are *link community* algorithms, while ELPA, LPA and COPRA are dynamic label propagation algorithms. ELPA and COPRA are both inspired by the LPA method. We compared ELPA with Linkcomm and LPA. Linkcomm clustered communities based on the similarity of edges, while ELPA discovered communities based on label propagation; Linkcomm is a hierarchical clustering algorithm, which needs a cut-off parameter, while ELPA is a heuristic method, and parameter free. Linkcomm is a highly overlapping method, while ELPA is a moderate overlapping method. Although ELPA is an extension of LPA in methodology, they have distinct differences. LPA is a node label propagation algorithm, while ELPA is designed for edge labels. LPA initializes each node to a unique community, while ELPA initializes a number of link communities (in most cases, it is half of the number of nodes). LPA starts a process with a random seed node, while ELPA begins with confirmed link communities. LPA depends on initial conditions and tie-break rules for its execution, while ELPA doesn’t. ELPA is a deterministic algorithm, while LPA isn’t; ELPA can discover overlapping communities, while LPA can’t. Moreover, ELPA includes edge clustering and node clustering. It uses edge clustering to check whether two vertices of each edge share label(s), while node clustering is similar to the main component of LPA.

We also compared ELPA with NeTA which is a node community algorithm previously developed by us. From Fig. 1 to Fig. 4 we see that ELPA can achieve comparable results to NeTA in most cases. NeTA is a local cohesive module detection method based on static network topology, while ELPA is a global condensed module discovery method that depends on dynamic edge label propagation and static network topology; NeTA can uncover smaller modules than ELPA in most cases, while as Table 2 shows, ELPA runs much faster than NeTA in most cases.

We have tested ELPA using different types of networks: social, online-social, collaboration and biological networks. For most of the real-world networks there is a lack of ground-truth, and therefore it is difficult to quantitatively evaluate the quality of community partitions or detected overlapping nodes. The best we can do is assesses the true structure using reasonable criteria. We compared ELPA with state-of-the-art methods previously reported in the literature by combining NMI, modularity and partition density, and we found that it preforms competitively with other algorithms on both synthetic and real-world networks.

## Methods

ELPA inherits the strengths of link communities with the efficiency of the label propagation algorithms (LPA) and it can discover both link communities and node communities. ELPA assumes that densely connected edges should form a consensus link community based on their edge labels, and that each edge (node) should update its label at each iteration step based on the labels of its neighbors. ELPA includes four main steps: initialization, edge label propagation, node label propagation and bridge identification.

### Initialization

**Figure 5:**
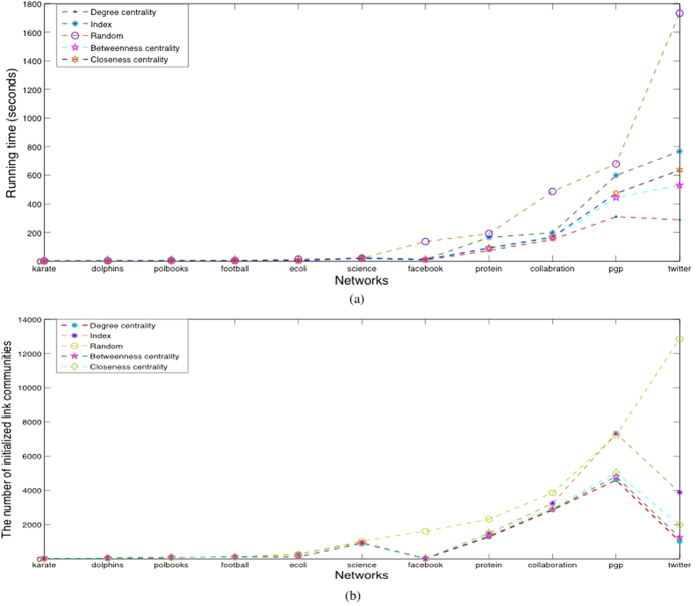
Comparison of initialization methods. (**a**) The number of initialized communities based on five initialization methods (degree centrality, indices of vertices, random, betweenness centrality and closeness centrality) the with the high-degree vertices. (**b**) Running time of the algorithm based on the five initialization methods.

Due to the observation that the edges that connect with high-degree vertices are more likely to form the ‘core’ part of one link community, we initialize link communities based on the high-degree vertices for a given network. In fact, the initiation also contributes to improve the convergence speed. As Fig. 5 shows, we use degree centrality to compare with other methods. In most cases, the number of initial communities generated by degree centrality is minimum, and the time complexity of the algorithm based on degree centrality is the lowest (the time complexities of computing the betweenness and closeness centrality are not taken into account). Thus initialization based on the high-degree vertices can decrease the number of initial communities, and reduce the running time of the algorithm in all the real-world networks effectively.

**Figure 6:**
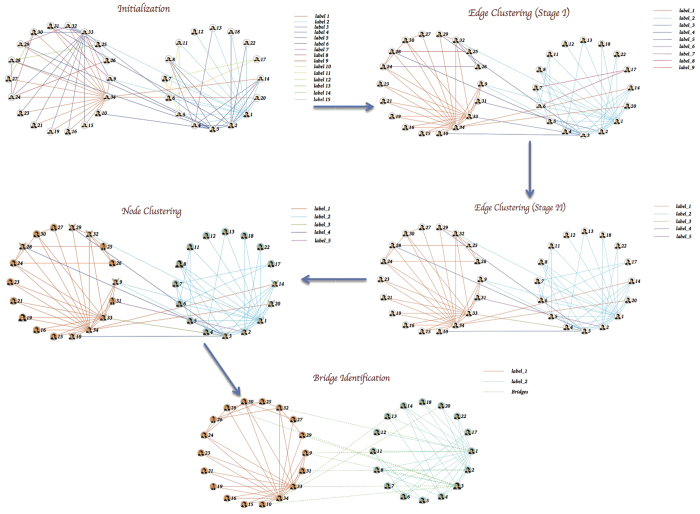
The illustration of ELPA based on Karate Club network. The colors of link communities are identical with the colors of corresponding node communities, and bridges are denoted with green dashed lines.

**Figure 7:**
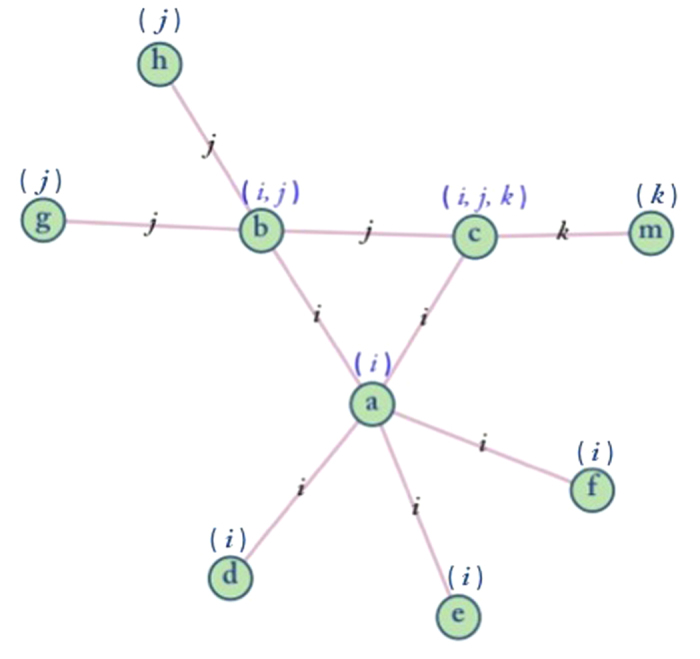
Diagram of the “triangle rule”. The edge between vertex *b* and *c* are labeled with the *j*^*th*^ link community, and vertex *b* and *c* has one common neighbor *a*. The label of vertex *b* is (*i*, *j*) and the labels of vertex *c* is (*i*, *j, k*), so they share one label *i*. Hence, We update the label of the edge between *b* and *c* to the *i*^*th*^ link community.

For example, in the karate network, the step of initialization is the following, the degree of vertices in descending order of degrees are 34, 1, 33, 3, 2 etc. respectively. Then we extracted all the edges connected with vertex 34, and took them as the first initialized link community; next we extract all the edges connected with vertex 1, and took them as the second initialized link community; and so on, until all the edges of this network are assigned to a certain link community. Finally, as Fig. 6 shows, we initialized 15 link communities from the karate network. A diagram of the initialized edge (node) labels are shown in Fig. 7, every edge is marked with a unique label, and every node is denoted by multiple edge labels.

### Edge label propagation

Edge label propagation includes two stages, stage I only performs edge clustering based on edge label propagation, while Stage II adds a trend label for each vertex before a standard edge label propagation process. In the process of edge label propagation, the iteration continues until no label changes.

Edge label propagation is processed based on the “triangle rule”. The main idea of the “triangle rule” is that if a pair of friends *b* and *c* have a common friend *a*, and furthermore both *b* and *c* are in the circle of friends of friend *a* (*i*^th^ link community), then the relation between *b* and *c* should be the intra relation of *i*^th^ link community. Thus we update the label for each edge based on this simple assumption. If there is more than one triangle that satisfies the “triangle rule”, which means vertex *b* and *c* share more than one link community. In this case, we update the label of *e*_*bc*_ (the edge between vertex *b* and *c*) to the one with the largest dimension among these shared link communities. For the edge *e*_*bc*_, at the *t*^th^ iteration it updates its label based on the labels of its adjacent edges at iteration *t*-1. Hence, *L*_*bc*_(*t*) = *f* (*L*_*ab*_(*t*-1), *L*_*ac*_(*t*-1)), where *L*_*x*_(t) is the label of edge *x* at time *t*. For example, As Fig. 7 shows, at time *t*-1, *L*_*bc*_(*t-*1) = *j*, vertex *b* and *c* share one common neighbor *a*, which means vertex *a*, *b* and *c* form a triangle. Because *L*_*ab*_(*t*-1) = *i* and *L*_*ac*_(*t*-1) = *i*, then *L*_*bc*_(*t*) = *i*, so we update the label of edge *e*_*bc*_ to the *i*^th^ link community as well. We perform this process iteratively, where at every step, each edge updates its label based on the “triangle rule”.

**Figure 8:**
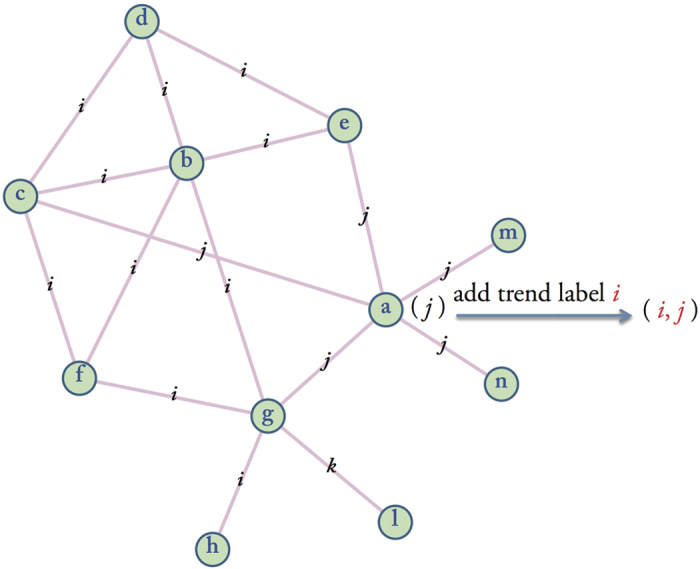
Diagram of add trend label. Vertex *a* only have a edge label *j* at time *t*-1, but most of the adjacent edges of its neighbors belong to the *i*^*th*^ link community, so we add a trend label *i* to the label of vertex *a*.

At stage I, the efficient edge labels of a vertex may be covered by other labels, so at stage II, we solve this issue by an adding trend label(s) approach. If most of the adjacent edges of its neighbors are concentrated in a unique label, then we take this label as its trend label. If not, we take the label(s) most of its neighbors joined in as its trend label(s). For vertex *k*, at the *t*^th^ iteration, it updates its label based on the labels of the adjacent edges of its neighbors at iteration *t*-1. Hence, ln_*k*_(*t*) = *f* (*L*_*k*11_(*t*-1), …, *L*_*k*1*l*_(*t*-1), …, *L*_*km*1_(*t*-1), …, *L*_*kmr*_(*t*-1)), where ln_*k*_(*t*) is the label of vertex *k* at time *t* and *L*_*ki*1_(*t*-1) …, *L*_*kir*_(*t*-1) are labels of adjacent edges of *i*^th^ neighbor of vertex *k* at time *t-*1. For example, As Fig. 8 shows, at stage I, label *j* covered all the adjacent edges of vertex *a* at time *t-*1, ln_*a*_(*t-*1) = *j*, but most of the edges of its neighbors belong to the *i*^th^ link community, which means vertex *a* lost efficient edge label *i*, then ln_*a*_(*t*) = *i*, so we updated its label by adding trend label *i* to its node label, the label of vertex *a* is set as (*i*, *j*) finally.

Figure 6 shows the edge label propagation clearly. At stage I, based on “triangle rule”, we updated the label for each edge of the karate network, and 9 link communities are left at the end this phase. While at the end of stage II, only 5 link communities are left.

### Node label propagation

Node clustering only processes node label propagation. After we have achieved edge clustering, many nodes still have multiple edge labels, so we need to further implement node clustering to discover node communities. The main idea of node label propagation is the following. Suppose that a node *k* has neighbors *k*_1_, *k*_2_, …, *k*_*m*_ and that each neighbor carries labels denoting the link community to which they belong to. Then *k* determines its community based on the labels of its neighbors. Hence, ln_*k*_(*t*) = *f* (ln_*k*1_(*t*-1), …, ln_*km*_(*t*-1)). That is to say, for each node *k*, if most of its neighbors share a unique label *i* (*i*^th^ link community), then *k* should join in *i*^th^ link community as well; if not, while most of adjacent edges of node *k* share a unique label *i*, then *k* should join in *i*^th^ link community as well. If none of the above conditions are satisfied, *k* should join in the link community(s) that most of the edges among its neighbors are concentrated in. Finally, most of nodes will belong to a unique community, and those nodes that still have multiple edge labels are denoted as overlapping nodes. For example, as Fig. 6 shows, at the end of this phase, each node only contains a unique label in the karate network.

### Bridge Identification

We mark one edge as a bridge if the labels of its two vertices are different. That is to say, for each edge, if its two vertices share label(s), it should be in the corresponding labeled link community, if not, this edge is a bridge.

For example, as Fig. 6 shows, at the end of this phase, the karate network is split into two link (node) communities. The two node communities detected by ELPA are identical to the observational study of Newman^32^. (A dispute between the club president and the instructor lead to the university sports club members splitting into two groups).

The following is the summary of proposed algorithms:

### Algorithm

For an undirected, un-weighted network, the main steps of ELPA are as follows:

1. Initializing the label (labels) for each edge (node) in the network, and set *t* = 1.
2. For each edge *x*, implement edge clustering based on the “triangle rule”, and update its labels *L*_*x*_(t).
3. For each node *k*, update its labels ln_*k*_(*t*) based on the labels of its adjacent edges.
4. Set *t* = *t* + 1, go to (2), until no label changes.
5. For each vertex *k*, updates its labels ln_*k*_(*t*) by adding a trend label, and then implement edge clustering again.
6. Set *t* = *t* + 1, go to (5), until no label changes;
7. For each node *k*, updates its label ln_*k*_(*t*) based on node label propagation to achieve node clustering.
8. Set *t* = *t* + 1, go to (7), until no label changes;
9. Check labels of the two vertices of each edge to mark bridges.
10. Go to (7), until no label changes;
11. If there are isolated communities or nodes, output them.

### Evaluation methodology

To evaluate the performance of a community detection algorithm, for those networks with ground-truth, we use the normalized mutual information (NMI) measure^17^ to evaluate the quality of a partition in the experiments reported in this paper. For the networks that lack a ground-truth, as the traditional G&N modularity measure is defined only for disjoint communities, we use the overlap modularity measure of ref. 41 for the experiments in this paper, which is an extended version of the overlap modularity defined by Nicosia *et al.*^42^.

### Complexity analysis

The time complexity of each step of the algorithm is roughly estimated below. Given a network with *n* nodes and *m* edges *N*(*n*, *m*), let *v* be the maximum degree of nodes in this network.

1. *Initialization* takes time *O*(*n*). Adding edge labels based on the node degree sequence takes time at most *O*(*n*), and adding labels for each of the *n* vertices takes time *O*(*n*).
2. *Edge label propagation* (stage I) takes time about *O*(*m* + *n*). This phase updates edge labels for each edge, it iterates through two vertices, which takes time *O*(*m*), and updates node labels for each node, which takes time *O*(*n*). This phase is repeated, until node labels no longer change, so the time per iteration is *O*(*m* + *n*).
3. *Edge label propagation* (stage II) costs about *O*(*vn* *+* *m*) time. This phase first adds trend labels for each node, which takes time *O*(*vn*), then, similar to phase 2, updates edge labels for each edge, which takes time *O*(*m* + *n*). Lastly, it updates node labels for each node, which takes time *O*(*n*). This phase is repeated, until link communities no longer change, so the time per iteration is *O*(*vn* + *m*).
4. *Node label propagation* takes time *O*(*nv*). For each vertex, it iterates through at most *v* neighbors, and the upper bound of cost time is *O*(*nv*).
5. *Bridge identification* takes time *O*(*m*). It iterates through all the edges and takes time *O*(*m*).
6. Phase 4 and 5 are repeated, so the time per iteration is *O*(*vn* + *m*).

As a result, the time complexity of ELPA is roughly *O*(*vn* + *m*).

## Additional Information

**How to cite this article**: Liu, W. *et al.* Discovering communities in complex networks by edge label propagation. *Sci. Rep.* **6**, 22470; doi: 10.1038/srep22470 (2016).

## Figures and Tables

**Table 1:** Real-world networks.

Networks	Type	Nodes	Edges	Reference
Karate	Social	34	78	28
Dolphins	Social	62	159	29
Football	Social	115	613	2,30
Polbooks	Social	105	441	2
E. Coli	Biological	418	519	31
Net-science	Collaboration	1461	2742	32
Facebook	Online-social	2888	2981	33
Protein	Biological	3274	8748	34
Scientific Co.	Collaboration	5242	14490	35
PGP	Social	10680	24316	36
Twitter	Online-social	23370	32831	37

**Table 2:** Running time comparison.

Networks	*T*_*ELPA*_(s)	*T*_*NeTA*_(s)
Karate	0.291250	0.884550
Dolphins	1.135768	0.670708
Football	3.803279	1.161884
Polbooks	3.809240	0.581783
E. Coli	2.789446	5.906084
Net-science	20.436553	251.00638
Facebook	7.038406	17.862583
Protein	73.268725	492.95475
Scientific Co.	149.690581	2011.7556
PGP	310.035794	6017.9896
Twitter	288.636866	5710.9980
